# Prolapsus Ani Successfully Treated by Pypodermic Injections of Strychnia

**Published:** 1881-02-20

**Authors:** Leonard Weber

**Affiliations:** New York


### CASE OF PROLAPSUS ANI SUCCESSFULLY TREATED BY HYPODERMIC INJECTIONS OF STRYCHNIA.

BY LEONARD WEBER, M. D., NEW YORK.

Nealton was the first, I believe, to recommend the use of strychnia for the cure of simple prolapsus ani. Whether he or any one else had used strychnia hypodermically for that purpose before I did, in 1868, I do not know.

In that year I was consulted by a merchant, about forty-five years of age, who had suffered from prolapsus ani for three years. It came on after a prolonged attack of dysentery. Not more than one inch of mucous membrane protruded.

It was easily reduced, but as readily came down again. Sphincter very weak and dilatable, but control over bowels satisfactory. At stool he would often lose small quantities of blood, and a slight but constant sero-sanguinolent discharge from the protruded mucous membrane was quite annoying to him. The usual remedies had been applied without success, and to the application of nitric acid, or the actual cautery, I could not persuade him to submit. It occurred to me to inject strychnia hypodermically. Inserting the needle about three-fourths of an inch from the anus, and directing it upward and parallel to the gut, I injected one-twelfth of a grain of the remedy, repeating the injection in forty-eight hours upon the opposite side, and continuing in this way until six injections had been made. The pain accompanying the injection was insignificant, no inflammation or abscess followed, the bowel ceased coming down, and the cure then effected has been permanent.

Case II. (1870).—Boy, eight years old, somewhat anæmic, muscular system poorly developed, had had repeated diarrhœal attacks. His mother said his "body" had been coming down for a long while. Prolapse half an inch. Sphincter very weak and dilatable. I injected one-eighteenth of a grain as above. The relief was complete after eight injections, given in the course of four weeks.

I have lost sight of this patient, and do not know whether the cure has been permanent.

Case III. (1877).—Boy, four years old, healthy and strong; prolapse of three-fourths of an inch, quite reducible, for about a year. Cure after four injections of one-twenty-fourth of a grain of strychnia, each given as above. Patient has remained cured.

Case IV. (1878).—Boy, five years old. No organic disease, but rather weak; troubled by frequent epistaxis. Prolapse nearly one inch long, in consequence of dysentery. Has had it for eighteen months, and been unrelieved by treatment so far. Four injections of one-twenty fourth of a grain of strychnia each were made, when the patient ceased coming to the office, and was lost sight of.

Case V. (1879).—Girl, six years old, somewhat anæmic, but well developed. Prolapse of half an inch, with considerable sero-sanguinolent discharge from the protruded mucous membrane, and occasional loss of blood at stool. It had existed more or less for two years, and had also followed dysentery. Cure after four injections of one-twenty-fourth of a grain of strychnia each. Patient has remained cured.

This was the only case in which I had to etherize the patient, owing to her excessive fear of being hurt. In all five cases the usual local and general treatment, tonic and astringent in character, had been tried without any benefit.

A speedy and permanent cure I know to have been obtained by the injection of strychnia, in *loco morbi*, in three cases. No pain of any consequence was inflicted by the procedure, nor unpleasant symptoms, inflammation or abscess, followed the injections. No such results have been obtained in my practice, in similar cases, by other remedies short of severe surgical measures.

It appears, then, from the record of these cases, that the hypodermic injection of strychnia in *loco morbi*, in cases of simple prolapsus ani, has a direct and rapid effect upon the sphincter muscles, re-establishing the physiological tone after comparatively few injections. This mode of treatment is perfectly safe, and apt to effect a speedy and permanent cure.—*New York Medical Record.*

